# Deep transcriptomic study reveals the role of cell wall biosynthesis and organization networks in the developing shell of peanut pod

**DOI:** 10.1186/s12870-021-03290-1

**Published:** 2021-11-03

**Authors:** Kapil Gupta, Shubhra Gupta, Adi Faigenboim-Doron, Abhinandan Surgonda Patil, Yael Levy, Scott Cohen Carrus, Ran Hovav

**Affiliations:** 1 Department of Field Crops, Plant Sciences Institute, ARO, Rishon Lezion, Israel; 2 Department of Biotechnology, Siddharth University, Kapilvastu, Siddharth Nagar, UP India

**Keywords:** Cellulose synthases, Cell Wall, Pod wart, Peanut, Shell development, Transcriptome, Transcription factors

## Abstract

**Background:**

Peanut (*Arachis hypogaea* L.) belongs to an exceptional group of legume plants, wherein the flowers are produced aerially, but the pods develop under the ground. In such a unique environment, the pod’s outer shell plays a vital role as a barrier against mechanical damage and soilborne pathogens. Recent studies have reported the uniqueness and importance of gene expression patterns that accompany peanut pods’ biogenesis. These studies focused on biogenesis and pod development during the early stages, but the late developmental stages and disease resistance aspects still have gaps. To extend this information, we analyzed the transcriptome generated from four pod developmental stages of two genotypes, Hanoch (Virginia-type) and IGC53 (Peruvian-type), which differs significantly in their pod shell characteristics and pathogen resistance.

**Results:**

The transcriptome study revealed a significant reprogramming of the number and nature of differentially expressed (DE) genes during shell development. Generally, the numbers of DE genes were higher in IGC53 than in Hanoch, and the R5-R6 transition was the most dynamic in terms of transcriptomic changes. Genes related to cell wall biosynthesis, modification and transcription factors (TFs) dominated these changes therefore, we focused on their differential, temporal and spatial expression patterns. Analysis of the cellulose synthase superfamily identified specific *Cellulose synthase* (*CesAs)* and *Cellulose synthase-like* (*Csl)* genes and their coordinated interplay with other cell wall-related genes during the peanut shell development was demonstrated. TFs were also identified as being involved in the shell development process, and their pattern of expression differed in the two peanut genotypes. The shell component analysis showed that overall crude fiber, cellulose, lignin, hemicelluloses and dry matter increased with shell development, whereas K, N, protein, and ash content decreased. Genotype IGC53 contained a higher level of crude fiber, cellulose, NDF, ADF, K, ash, and dry matter percentage, while Hanoch had higher protein and nitrogen content.

**Conclusions:**

The comparative transcriptome analysis identified differentially expressed genes, enriched processes, and molecular processes like cell wall biosynthesis/modifications, carbohydrate metabolic process, signaling, transcription factors, transport, stress, and lignin biosynthesis during the peanut shell development between two contrasting genotypes. TFs and other genes like chitinases were also enriched in peanut shells known for pathogen resistance against soilborne major pathogens causing pod wart disease and pod damages. This study will shed new light on the biological processes involved with underground pod development in an important legume crop.

**Supplementary Information:**

The online version contains supplementary material available at 10.1186/s12870-021-03290-1.

## Background

Peanut (*Arachis hypogaea*) belongs to an exceptional group of legume plants, wherein flowers are produced aerially, but pods develop underground. Upon fertilization, a specialized organ called the gynophore (peg) elongates and grows downward, pushing the fertilized ovary into the soil. Inside the ground, the gynophore undergoes robust changes during development. In the early stages, the swelling gynophores have root-hair-like structures that function to absorb water and nutrients from the ground to benefit the mother plant [1]. Later, gynophore tips transform into pods, fruit structures that originated from the carpel. The developing pod no longer has a root-like function, but can absorb water and nutrients like Ca^2+^, essential for normal seed development through the outer shell [2].

To fully mature, the developing pod requires three principle conditions: complete darkness, soil friction, and moisture [3]. In such an environment, the pericarp plays a vital role as a barrier against mechanical damage and soilborne pathogens. Shell also serves as the intermediate source of carbohydrates and amino acids through the plant source (leaves) to the developing seed sink (embryo) as well as producing metabolized storage products [4, 5]. Like other legumes, sucrose acts as a temporary reserve form of carbohydrates in the shell [6]. At the start of peanut pod development, the shell wall or pericarp occupies most of the pod’s volume. Later, the expanded seeds gradually increase in the shell with the pod development [7]. Boote [8] divided this peanut pod development process into five distinct stages: pods with tiny embryos (R4), seed growth (R5), fully expanded but immature seeds (R6), fully developed and fully mature wet seeds (R7), and mature dry seeds ready for commercial use (R8).

The peanut shells’ chemical composition and physical properties vary vastly during pod development [9]. In the young pod, the soft tissue surrounding the embryo contains sucrose, starch, and water; however, towards maturation, fibrous hemicellulose content increases, and other non-fibrous components decreases [10]. The mature shell/pericarp contains mainly cellulose, hemicelluloses, lignin, and pectins as the crosslinking agents [11]. Chemical composition of shell is 8.2% protein, 28.8% lignin, 37.0% cellulose and 2.5% carbohydrate [12].

Besides pectic compounds, Ca^2+^ can form complexes in the cell wall of the peanut shell by crosslinking with lignin or carbohydrates via the phenolic acids and play a vital role in the stabilization of the cell wall [13, 14]. Ca^2+^ distribution was found to vary considerably during the development of peanut shells; higher in the pectin fraction at the beginning of enlargement (R5), an enlargement in the lignin fraction (R6), and at the expanded point (R7) in the cellulose fraction [15]. Ferulic acid is another stabilizing agent of the cell walls [16], whose residues are attached to glucurono arabinoxylan (GAX) and may serve as nucleation sites for lignin formation [17, 18]. Apart from structural constituents, other biochemicals like flavonoids are also present in the peanut shells. Pendse et al. [19] isolated three flavonoids-5,7-dihydroxychromone, eriodictyol, and luteolin; the latter accumulates during the shell’s maturation and changes the shell’s color from yellow to orange and orange to brown towards reaching maturity [20]. Therefore, pod shell components in peanuts are unique compared to other legume pods, and it resembles cell wall biogenesis and wood formation in tree plants.

Besides their biological role, peanut shells also have several industrial usages. The peanut shell’s fibrous/polysaccharide content makes it a suitable substrate for hydrothermal decomposition to recover reducing sugars and value-added compounds [21], fuels [22], animal fodder, and for livestock bedding. The biogenesis and shape of the shell often play an important role in the ‘in-shell’ peanut industry, wherein the peanuts are processed, roasted, and marketed with their outer shells. The appeal of this business relies on the appearance of the shells, and consumers want shiny, bright shells without stains or diseases.

Few recent studies have reported about uniqueness and importance of gene expression patterns that accompany peanut pods’ underground biogenesis and development. In the report on early developing peanut pods, they identified changes in light signaling, hormone biosynthesis/signaling, and downstream changes in cell division and cell wall development [23]. Chen et al. [24] reported hundreds of significantly changed transcripts associated with gravitropism and photomorphogenesis processes during pod formation. An atlas for peanut developmental transcriptome was also published [25], wherein an enrichment for cell wall and secondary cell wall, photorespiration, copper ion binding, protein folding and translation processes were reported to be enriched in the developing pod. Involvement of the processes like cell wall, transport, stress, transcription, signaling, UDP-D-xylose biosynthesis and UDP-sugars interconversion are related to cell wall synthesis and pod thickening during pod expansion/enlargement [26]. Other studies have reported the involvement of hormone-responsive genes in the pod development and maturation, like auxin-induced proteins, gibberellin beta-dioxygenases, ethylene-responsive transcription factor, cytokinin hydroxylase, cytokinin dehydrogenase, abscisic acid insensitive 5-like protein and abscisic acid hydroxylase [27, 28].

Application of combined studies of transcriptomic, proteomic and miRNA during pod formation in peanut has described the role of gene-miRNA in this process [28, 29]. Epigenetic regulation of peanut pod development mediated by methylation has expanded our understanding of the pod development process [30]. These studies provide an excellent glance at the pod development-related processes in peanuts but they limited their focus on the formation of the peg and early pod stages. Late development stages still need to be addressed in which the pod also develops resistance against the soil-borne pathogens.

In the current research, gene expression analysis was carried out during pod development in two peanut genotypes that vary greatly in their shell characteristics to expand this knowledge across more defined developmental time frames and in a more comparative manner. Transcriptome analysis of pericarp (shell) development was performed in pods utilizing a time series of four developmental stages using gene annotation details from the sequenced genomes [31, 32] as well as other tools [25, www.peanutbase.org]. Metabolite analysis was also performed to get significant stage-specific changes in the shell’s components. The present study’s primary focus is on cell wall biosynthesis and the structural processes crucial for identifying genes involved in reorganization, development, and stress resistance. Therefore, this study dealt with the cell wall organization in both early and late pod development stages that build the barrier against abiotic and biotic stress. Identified TFs related to pathogen resistance can play key players in the development of pathogen-resistant peanuts for ‘in shell’ market segment.

## Results & discussion

### Transcriptomics study of two peanut genotypes during shell development

Two peanut genotypes, Hanoch and IGC53 (the local name for PI3383), that differ from each other based on shell properties (pod size, pod-filling potential, shell structure, and disease resistance) were studied. To underlay the difference in transcriptional reprogramming during shell development four developmental stages of the peanut shell, i.e., R4, R5, R6, and R7 were selected from both genotypes, and RNA-Seq libraries were prepared. After the cleaning procedure, an average of 13.6 million reads per sample was extracted for each library, and approximately 97% of the reads were mapped to the genome-guided transcript assembly. The total expression counts for the 120,364 peanut transcripts were measured, of which 60,814 transcripts belong to Agenome (*Arachis duranensis)* and 59,551 transcripts to B-genome (*Arachis ipaensis)*.

**Fig. 1:**
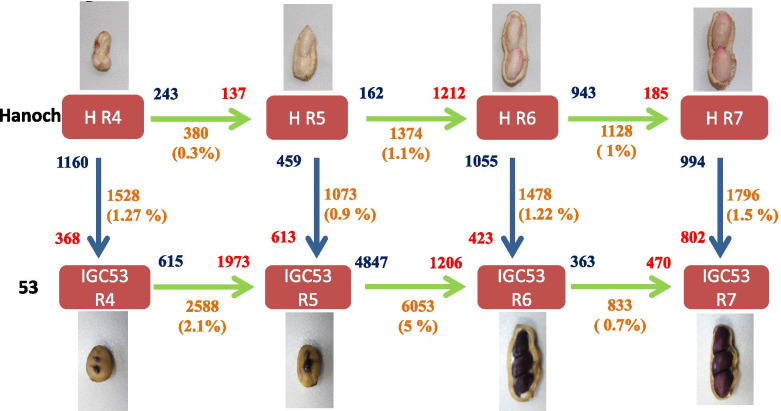
Differential expression of genes during pod developmental stages, between and within genotypes Hanoch and IGC53. Comparison between Genotypes is shown with Blue arrows and between development stages of a genotype by Green arrows. The total number of DE genes are shown at middle of the arrow; down or up regulated genes, have been shown in blue and red color respectively

The dynamics of shell development in terms of gene expression were compared between genotypes during the developing stages (Fig. 1). Generally, the number of DE genes was higher in IGC53 than in Hanoch. During development, the R5-R6 transition was the most dynamic in terms of transcriptomic changes (1.1 and 5% genes differentially expressed in Hanoch and IGC53 respectively), then R4-R5 (0.3% in Hanoch, 2.1% in 53), and R6-R7 (1% in Hanoch, 0.7% in IGC53) (Fig. 1). This difference in gene expression during the R5-R6 transformation corresponds to the phenotypical difference between these two stages, since in R5 the shell is still smooth and soft, while in R6 the shell starts to become more rigid. Yet, IGC53 has a more rigid and reticulated shell in general, and the reticulation is developed earlier than in Hanoch. This can explain the up-regulation of higher number of genes in IGC53, particularly in early and medium developmental stages.

### Enriched biological processes during peanut shell development

**Fig. 2:**
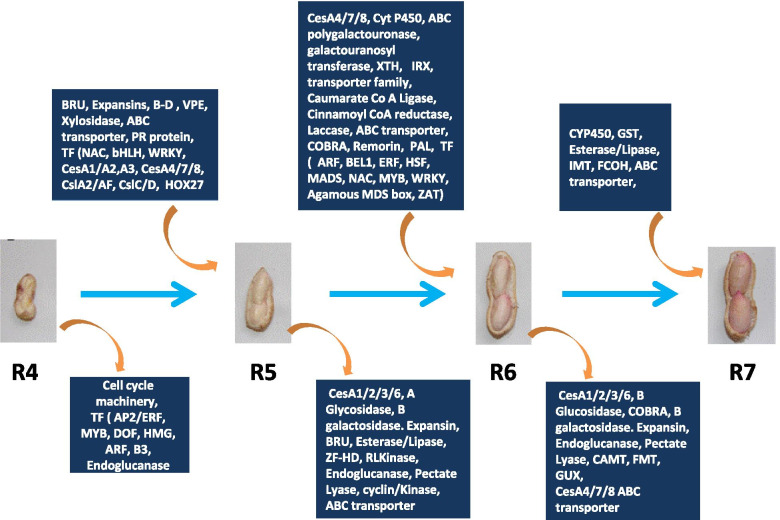
Graphic representation of genes involved in the shell development process. The list of genes is extracted from the GO analysis for processes/activities during pod development (Supplemental File 1). Rounded arrows represent up-regulated and down-regulated genes in each developmental transition

Functional analysis of the DE genes during the stage transition was performed to understand the processes and activities that occur and change during successive developmental stages in both genotypes’ peanut shells (Supplementary file 1). An example of selected genes from these processes/activities that were up- or down-regulated during development in both genotypes is presented in Fig. 2. The transition from R4 to R5 developmental stage is witnessed with a small pod with tiny ovules to the enlarged pod with a real seed. Downregulation of DNA binding activity and TF activity was common in both genotypes during this period. Transporter activity, biosynthetic process and fruit ripening process (which includes Cytochrome P450 71A1s) were downregulated in Hanoch. In contrast, extracellular regions, cell cycle, cytoskeleton, and lipid binding processes were downregulated in IGC53.

Processes up-regulated during the transition from R4 to R5 included TF activity, proteinaceous extracellular matrix, structure morphogenesis, carbohydrate metabolic process, hydrolase activity, secondary metabolic process, and cell wall in both genotypes. This is not surprising since during this transition from R4 to R5 a significant change in pod enlargement occurs. The enlargement is assisted by the carbohydrate metabolic process especially cell wall biosynthesis. That included the up-regulation of genes like *Cellulose synthases* (*CesAs*), *Cellulose synthase*-*like* (*Csl*), *Brassinosteroid regulated proteins BRU1*, *Xyloglucan Endotransglucosylase*/*hydrolase* and *Expansins;* (Fig. 2) which play an important role in the expansion of the cell wall. BRU1 possesses xyloglucan endotransglycosylase (XET) activity [33], which mediates signaling in cell wall biosynthesis, while expansins unlock the network of polysaccharides and allow turgor-driven cell enlargement [34, 35]. Liu et al. [26] reported that the majority of the transcripts that up-regulated during pod expansion were related to the cell wall, transport, stress, transcription, cell cycle, cell organization and signaling. During pod enlargement, UDP-D-xylose biosynthesis and UDP-sugars interconversion were significant processes responsible for cell wall synthesis and pod thickening [26].

The transition during R5 to R6 is less characterized by pod enlargement but changes in shell reticulation and rigidness. The processes observed to be downregulated during R5 to R6 transition were cell wall, carbohydrate-binding, carbohydrate metabolic process, kinase activity, and extracellular region in both genotypes. Cell growth, cell cycle, kinase activity, and catabolic process were observed to be downregulated in IGC53 only. Downregulation of the carbohydrate metabolic process during this transition includes cellulose synthases (CesAs) 1, 3 and 6, which can be related to the completion of normal cell wall biosynthesis requirement in the pod. Another effect of the downregulation of cellulose synthases is the necessity for a secondary cell wall controlled by CesA4, 7, and 8. The processes observed to be up-regulated in the shell during the transition from R5 to R6 were transporter activity, TF activity, and secondary metabolic processes in both genotypes. Transport activity was overrepresented in the pod wall, also known to supply nutrients to the seed as part of the source-sink pathway [5]. Other processes observed in Hanoch were plasma membrane, abscission, proteinaceous extracellular matrix, and carbohydrate metabolic processes that overlap with secondary metabolic processes. The process of abscission corresponds here to COBRA family proteins. COBRA-like protein brittle culm1 (BC1) in Arabidopsis regulates secondary cell wall formation by cellulose formation/crystallization [36, 37], and suppression of BC1 led to the mechanical loss of stem and dwarf phenotype in rice [38]. Among the secondary metabolic process, major candidate genes like *4-Coumarate-CoA ligase 1 (CL)*, *Caffeoyl-CoA O-methyltransferase* (*COMT*), *Cinnamoyl-CoA reductase 1 (CCR1)*, *Transparent Testa,* and *Laccase* were expressed. These genes mediate the lignin biosynthesis pathway in developing pods, leading to solidifying the pod cell wall and a nondegradable barrier for pathogens, enhancing its protective effect against biotic stresses in soil [39, 40].

The transition from R6 to the R7 developmental stage represents the stage of reaching maturity, where further expansion is not needed, and the shell needs to lose water and accumulate nutrients to make the pod drier to maintain the mature stage and protect the seeds. During this transition, the processes downregulated are cell wall, extracellular region, carbohydrate metabolic process, and carbohydrate-binding. Response to external/biotic stimulus (*CesA 4*, *7* and *8*), abscission (*COBRA-like protein*) and cellular growth component processes were observed to be downregulated in Hanoch but not in IGC53. These processes can be related to the formation of a more compact organized shell in IGC53 that could produce resistance against pathogens compared to Hanoch.

The processes that were up-regulated during the transition from R6 and R7 matched the changes in shell maturation, water loss and protection. These included secondary metabolic processes, TF activity, fruit ripening, catalytic activity, biosynthesis processes, pollen-pistil interaction and lipid metabolic processes. The secondary metabolic process is represented by genes like *Isoflavonemethyl transferase*, *Cinnamyl alcohol dehydrogenase* (*CAD*) and *Feruloyl hydroxylase* that represents lignin biosynthesis. At this stage, the involvement of *CYP450* was also observed that mediate metabolites modifications to synthesize monolignol for lignin biosynthesis. Another important and unique process observed is pollen-pistil interaction; represented by *G-type lectin S-receptor-like serine/threonine-protein kinases* (*RLKs*), which can be related to disease resistance. Seventy-two different *RLKs* were reported to be important candidates against leaf spot resistance in peanut [41]. *RLKs* function as pattern recognition receptors (PRRs) in pathogen/microbe-associated molecular patterns at the cell surface to activate a pattern-triggered immunity (PTI) response [42]. Higher numbers of *G-type RLK* genes were differentially expressed in IGC53 than Hanoch, which can be linked to better resistance against pathogens in IGC53.

The total percentage of DE genes observed during the shell’s development stage transition was relatively low compared to the seed development of the same two genotypes, previously reported by Gupta et al. [43]. The lower percentage of DE genes maybe due to the fact that shell does not imply as many complex processes during growth as the seed does, and is only limited to processes such as cell wall, extracellular matrix, cell division, TF activity, carbohydrate metabolic processes, response to biotic/external stimulus, and catalytic activity. Also, the genes in peanut shells were enriched for responses, against fungus, bacterium, and nematode; soilborne major pathogens [44, 45].

### Differentially expressed genes between Hanoch and IGC53 genotypes during shell development

**Fig. 3:**
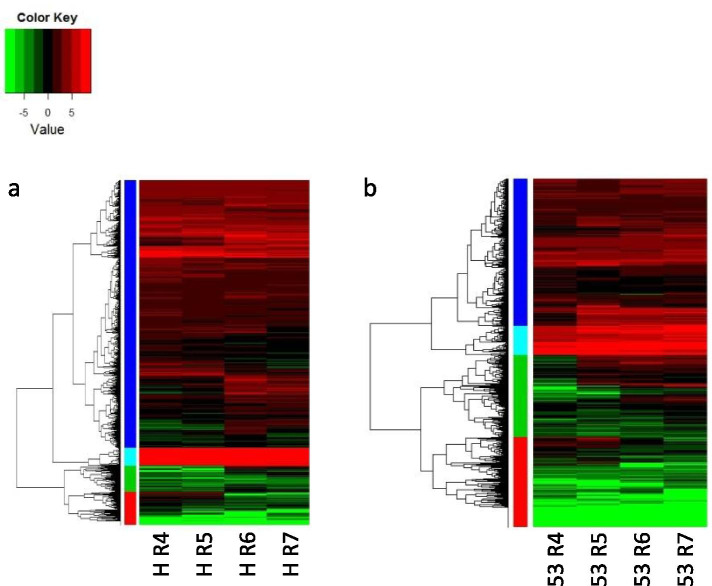
Cluster analysis of differentially expressed genes (DE) during peanut shell development (**a**) Hanoch and (**b**) IGC53

At each developmental level, differentially expressed genes were identified between Hanoch and IGC53 pods to further explore genotype-specific transcriptomic dynamics (Fig. 1). The most significant difference between Hanoch and IGC53 was observed at R7 and R4 stages, with 1796 and 1528 DE genes, respectively. In R6 and R5 developmental stages, 1478 and 1073 DE genes were found, respectively. 3301 DE genes between Hanoch and IGC53 during the pod development were subjected to hierarchical clustering based on mean expression and expression pattern, leading to four clusters for each genotype (Fig. 3). Subsequently, a transition matrix was performed to study the partitioning between the two genotypes to understand the differences and similarities during development. Shared genes between the clusters of Hanoch and IGC53 were recognized and presented in a 4 × 4 transition matrix and further analyzed for significant enrichment of GO terms (Supplementary File 2, Supplementary Fig. 1).

First, the cells in the transition matrix representing the same expression pattern in Hanoch and IGC53 were analyzed (the blue diagonal cells in Fig. 3). The high expressing cell in both genotypes (H-1:53–1) is related to processes like cytokinin binding, microtubule-based movement and lipid transport, connected to the organization of tissue and growth. The cell H-1:53–1 represents high expressing genes in both genotypes. It is enriched with processes like cell wall/secondary cell wall, lignin biogenesis, xyloglucan transferase activity, xylulose biosynthesis, and response to peroxide and vacuole. The H-2:53-2 cell represents processes related to cell wall development and processes responsible for modifying cell development. Another matrix with a similar expression cell, H-3:53–3, represents enriched processes for sulfate transport, ATP biosynthesis, flavonoid biosynthesis, alkane monooxygenases, suberin synthesis, serine carboxypeptidase activity, and cellulose synthases. Another similar expression matrix cell, H-4:53-4, represents a very low expression level in both genotypes and includes the genes responsible for GA-mediated signaling, peroxidase activity, extracellular region, sugar response and mannose-binding and NADH activity.

The transition matrix’s cells containing clusters expressing higher in Hanoch (shown as red cells in Supplementary Fig. 1) had a higher number of genes than cells containing clusters expressing higher in IGC53 (shown as green cells in Supplementary Fig. 1). The cells H-1:53-2, H-2:53-–3 and H-3:53-4 represent the processes expressed higher in Hanoch. The processes for energy generation, cell wall biosynthesis, flavonoid metabolism, transporter activity, chloride channel, potassium channel, and lipid response were overrepresented in these cells. ATP biosynthesis, electron carrier activity, and energy generation processes were also observed to be higher in Hanoch seeds than IGC53 seeds in a former study [43]. The character that mostly differentiates Hanoch from IGC53 is the pod size. Processes such as energy generation, cell wall biosynthesis and transporter activity may be related to the rapid enlargement of the Hanoch pod.

The cells H-2:53-–1, H-3:53-2, H-4:53-2 and H-4:53-3 represent the processes that are expressed higher in 53. Cell H-2:53-1 was over-represented by a group of genes that belong to TF activity and cellulose biosynthesis process. The presence of five TFs characterizes this cell, three of which code for AtHB13, one ZAT10, and one WRKY TFs. (Supplementary File 3). The HB13 codes for a leucine zipper homeodomain TF involved in the crosstalk between abiotic and biotic stress resistance [46]. HB13 TF was also observed to induce chitinase and glucanases in transgenic *Arabidopsis* plants (Cabello 2012). The chitinase and glucanase genes are located in the same H-2:53-1 cluster and could be responsible for the higher pathogen resistance of genotype IGC53 compared to Hanoch (Supplementary File 2). ZAT10 and WRKY30 are involved in abiotic stress tolerance in Arabidopsis and wheat [47–49].

### Expression study of cellulose synthase superfamily during Shell development

Due to their presumable importance, the cellulose biosynthesis and TF activity processes were further analyzed by focusing on the specific genes involved in these processes and their role in shell development. The *Cellulose synthase A* (*CesA*) and *Cellulose synthase-like (Csl)* gene families constitute cellulose synthase superfamily [50, 51] characterized by glycoside transferase activity with a class-specific region (CSR) and four conserved motifs (QxxRW, DD, DCD, and TED) known for substrate binding [52]. Nucleotide sequences of Arabidopsis *CesA* and *Csl* genes were used to identify the particular gene family in peanut using the local tBlastx program. A total of 155 genes of the cellulose synthase superfamily were identified, among which 69 genes were classified as *CesAs* and 86 as *Csls* (Supplementary File 4).

**Fig. 4:**
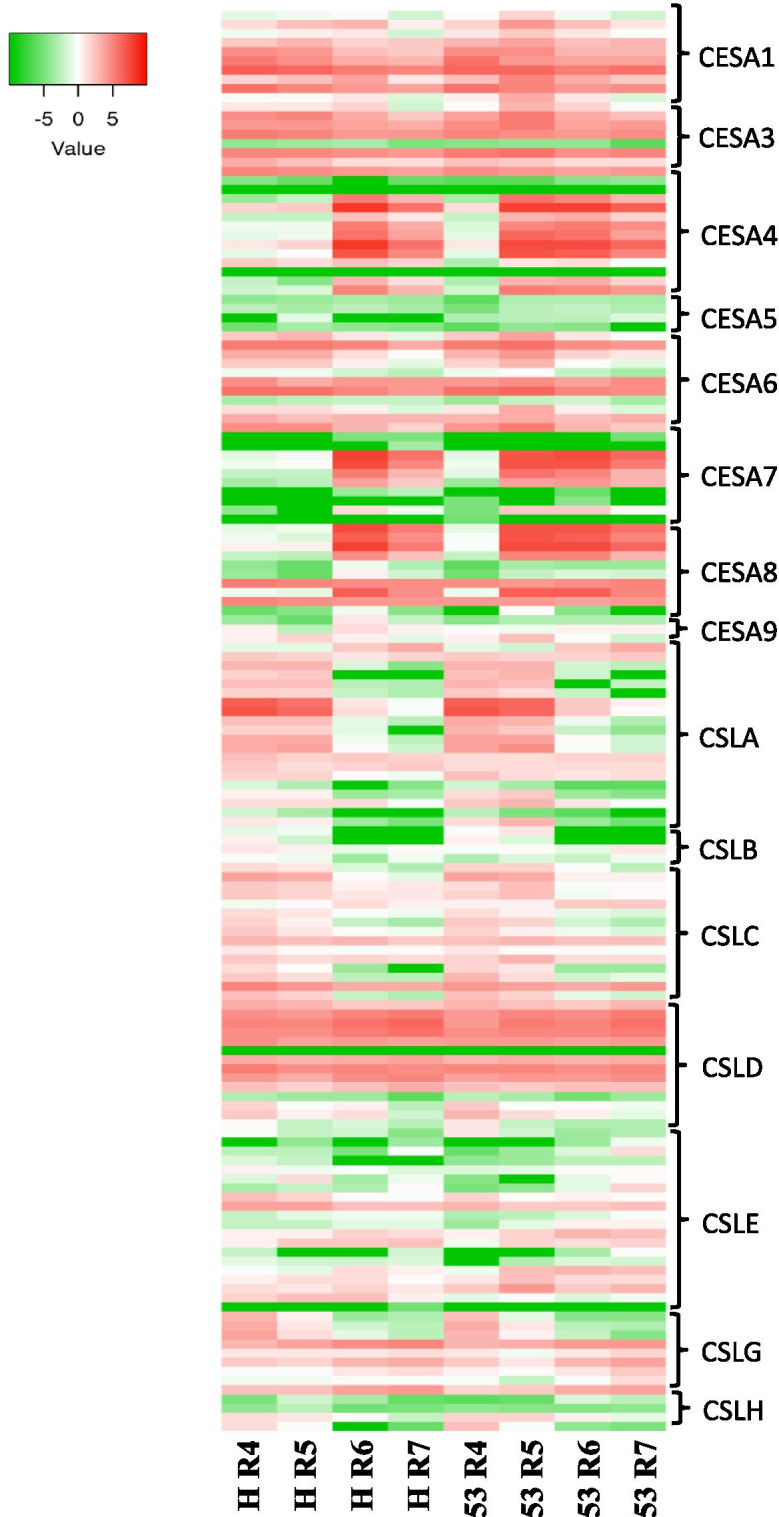
Expression of Cellulose Synthase Superfamily genes: Cellulose Synthase As (CesAs) and Cellulose Synthase-like (CSL) genes in peanut genotypes Hanoch and IGC53 during shell development

Based on specificity, the CesAs family can be categorized into two subgroups. I) *CesA* genes related to primary cell wall biosyntheses, like *CesA1*, *CesA2*, *CesA3*, *CesA5,* and *CesA6* [53]. II) *CesA* genes related to secondary cell wall biosyntheses like *CesA4*, *CesA7*, and *CesA8* [54]. log2 transformed RPKM expression of Ces superfamily genes was analyzed during different shell development stages (Supplementary File 5). Most of the members of *CesAs* are either expressed at a very low level or not expressed at any of the developmental stages of the shell; their role may be expected in vegetative organs or at specific tissue development. The Ces superfamily genes expressed at high or moderately high levels are expected to be related to the shell’s development. Expression of subgroup I *CesA* genes was observed uniformly high at all stages. In contrast, subgroup II *CesA* genes showed selective upregulation at R5 or R6 stages until maturity. Also, the group I *CesA* genes’ expression was almost similar in both genotypes, while group II members differed in their expression; higher in IGC53 at stages R5 to R7, but high in Hanoch at R6 and R7 (Fig. 4; Supplementary File 5).

The CesA family expression pattern in the shell was observed different from that previously found for seeds [43] (Supplementary File 5). Group I *CesA* genes’ expression pattern was similar during both shell and seed during development, while group II CesA genes were expressed at shell development stages but very low or negligible in the seeds. This observation suggests that cell division and primary cell wall biosynthesis continues from early stages till maturation in pod development. In contrast to this, secondary cell wall biosynthesis activates at later stages of development, leading to the shell’s hardness to protect developing seed underground.

Apart from the CesA family, Ces related cellulose synthase-like (Csl) gene family is also involved in cell wall biosynthesis. The *Csl* genes are mainly responsible for the biosynthesis of hemicelluloses [55] which along with cellulose, form a matrix in the cell wall [56]. *Csl* gene family in peanut are categorized in seven groups *CslA*, *CslB*, *CslC*, *CslD*, *CslE*, *CslG* and *CslH*. *Csls* code for β-Glycan synthase/glycosyltransferase enzymes, which play an important role in producing non-cellulosic polysaccharides (heteromannans, xyloglucans and 1,3; 1,4 β-glucans) in plant cell walls [57]. *CslA* codes for Mannan Synthase, *CslC* for Xyloglucan Synthase and *CslD* for Mannan synthase, while *CslF* and *CslH* genes encode mixed linkage glucan synthases [58, 59].

Expression of *CslA1* and *CslA2* are specific to the R4 and R5 stages, among which *CslA2* expression is comparatively higher than *CslA1*. Members of the *CslD* group, *CslD2* and *CslD3* are expressed at all developmental stages, while the *CslD5* expressed moderately high during early developmental stages (R4 and R5). *CslD2* and *CslD3* genes express at a high level and may synergistically work in a complex [60]. *CslC* members have been observed to play a role in xyloglucan biosynthesis and code for probable xyloglucan xylotransferase [61] for the synthesis of the (1,4)-β-glucan backbone of xyloglucans. *ClsC* group members’ expression during shell development showed to be moderately high and categorized into two types - one with continuous expression at all stages and the other with expression at only early stages (R4 and R5) (Supplementary File 5).

*CslB* group members either expressed very low or not expressed during development, suggesting no involvement in shell development. Indeed, the expression of *CslB* genes was reported to be limited to flower sepals or roots [62]. *CslH* group members’ expression remains low or negligible. *CslG1* is expressed at an early stage of the shell (e.g., R4), while *CslG2* members are expressed during all developmental stages. Expression of these genes was confined to peanut shells and not in the seeds, suggesting their importance in shell development.

### Expression study of genes involved in modifications to cell walls

Modifications in cell walls mediated by various cell wall modifying enzymes help the plants to adjust against environmental changes and control the entry of biotic agents. The architecture of cell walls is an important determinant of plant tolerance to multiple biotic stresses. An impenetrable, physical barrier formed by a rigid cell wall, protects against pathogen invasion [63]. Peanut shell is made up of cellulose crosslinked by non-cellulosic polymer hemicelluloses. Hemicellulose biosynthesis requires heteromannans, xyloglucans (XyGs), heteroxylans, and mixed-linkage glucans (MLG).

Six hundred and five genes related to the cell wall modifications process were identified from the GO term “Cell wall” and were specifically studied for their role during shell development. Few of them are *Endochitinase*, *Xylosidase* (*XS*), *Xylosyltransferase* (*XT*), *COBRA* (*COB*), *Xylose symporter* (*XSYM*), *β-Xylanase*, *Expansins* (*Exp*), *Xyloglucan Endotransglycosylase* (*XET*), *Xyloglucan Galactosyltransferase* (*XGT*), *Xylose Isomerase* (*XI*), *Xylosyltransferase Hydrolase* (*XTH*), *Chitinase* (*CHI*), *Xylose Kinase* (*XK*) and *Endoglucanases*. Some of the above like *Expansins*, *Xyloglucan Endotransglycosylase*/*hydrolase* and *endo*-(*1,4)*-*β-d-glucanase* are responsible for cell enlargement and expansion [64]. In regulating cell wall plasticity/rheology, other cell wall-modifying enzymes such as Pectinesterase (PE) and Polygalacturonase (PG) play a significant role.

**Fig. 5:**
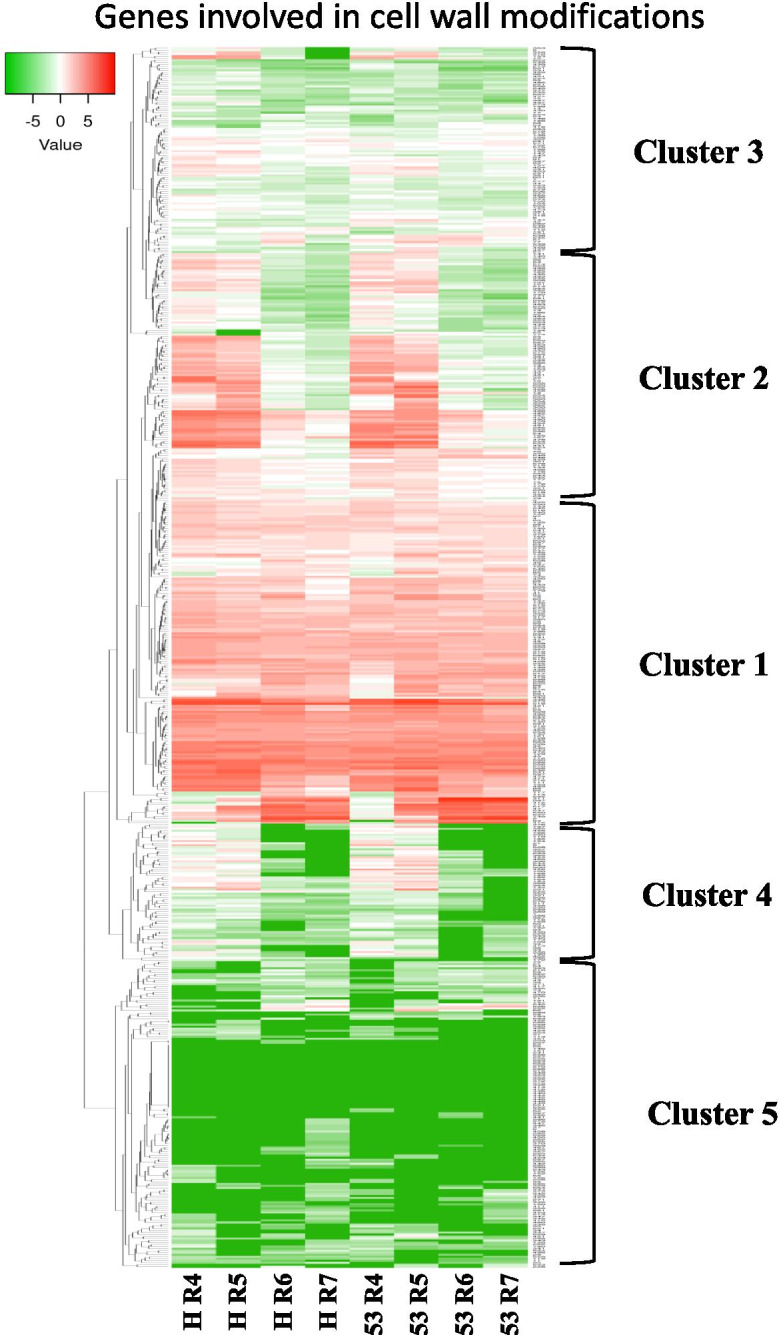
Expression analysis and clustering of genes involved in cell wall modifications in Hanoch and IGC53 peanut genotypes during shell development

Cluster analysis of cell wall modification-related genes revealed five clusters of expression patterns (Fig. 5; Supplementary File 6). Cluster 1 represents a very high expression pattern in all shell developing stages. It mainly involves genes like *XK*, *XT*, *XI*, *XGT*, *XET*, *Brassinosteroid-regulated protein BRU1*, *COBRA*, *XSYM*, *Glucan endo-1,3 beta-glucosidase 8*, *PG* and *PES*. Cluster 2 contains genes that are expressed in the early developmental stages (R4 and R5). Based on the expression analysis, genes that may be involved in cell wall modification are auxin binding protein, *XGT*, *XET*, *XK*, *XT*, XTH, *XI*, *BRU1*, *COBRA*, *XSYM*, *glucan endo-1,3 beta-glucosidase 008*, *EXP*, *Peroxidases*, *PG*, *PE*, *COBRA*, *Endochitinases PR4* and *Alpha XS*. High expression of *XTH*, *EXP*, *PG*, *PE* and *BRU1* proteins may be associated with an increase in the degree of cell wall plasticity and extension. The majority of these genes belong to xyloglucan (XyG) biosynthesis and modification enzymes, which serve as a spacer molecule required to prevent the microfibrillar formation of cellulosic aggregates [65, 66]. XTH transglucosylase activity helps integrate newly secreted xyloglucans into the cell wall to strengthen it [67]. Several *XHT*-encoding genes were up-regulated during early pod development and *EXP* were active during peanut pod development [23]. Genes encoding pectinesterases were up-regulated in expanding pods compared to initial pods [68]. A *COBRA* protein co-expressed with cellulose synthase, also co-localizes to cellulose synthase complex on the plasma membrane, acts as a “polysaccharide chaperone” to facilitate cellulose crystallization from the emerging 1–4 glucan chains [37], and it may help the peanut shell in providing the mechanical strength. Therefore, the cell wall modifying genes play an important role in restructuring the backbone of the polysaccharide in the peanut shell’s cell wall that protects the seeds against abiotic stress and biotic stress from soilborne pathogens.

### Expression analysis of transcription factors (TFs) in the shell development process

Another major process observed during peanut shell development was Transcription factor activity. Therefore, a focused study was done to identify significant TF regulators involved during peanut shell development. Three thousand twenty-one genes were identified to code for TF and were categorized into 67 TF families. The bHLH, bZIP, NAC, MYB, AP2/ERF, C2H2 and WRKY TF families were the most abundant TFs in the transcript assembly (Supplementary File 7). Most of the TF showed stage-specific expression in R5, R6 and R7, which correspond to their involvement in shell development (Supplementary File 7). MYB and NAC TFs were identified as major regulators of shell development processes as their expression was induced during the mid to end of the developmental stages. Expression variation of MYB and NAC TFs among genotypes is also observed, with a prolonged duration in IGC53 (from R5 to R7 stage) than Hanoch (R6 to R7 stage).

**Fig. 6:**
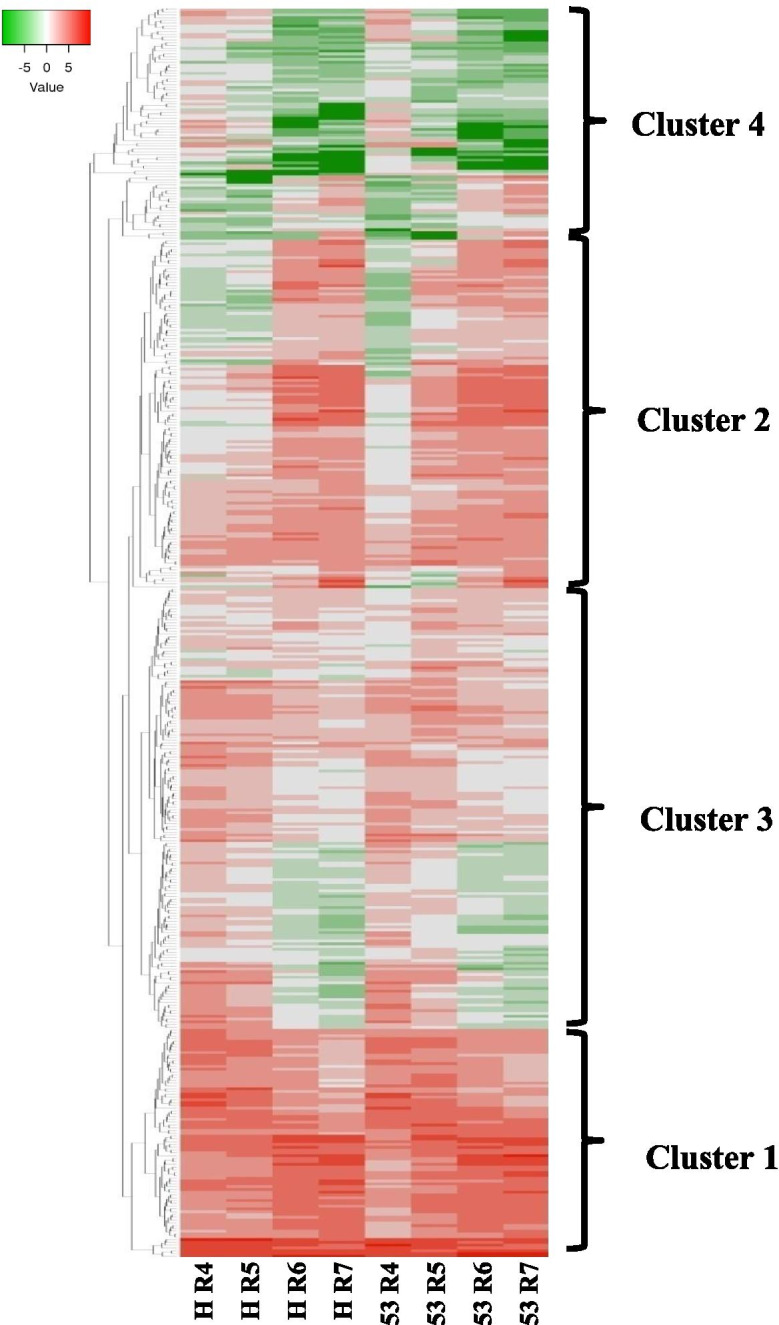
Expression analysis and clustering of 449 DE TFs during the shell development stages (R4-R5-R6-R7) in two peanut genotypes, Hanoch and IGC53

TFs that DE during any stage of peanut shell development in Hanoch or IGC53 were mined and pooled to study their expression using log_2_ RPKM values (Supplementary File 7). A total of 449 TFs were found to DE during the stages (R4-R5-R6-R7) of shell development irrespective of genotypes corresponded to the members of AP2/ERF, ARF, AUX/IAA, bHLH, bZIP, C2H2, DOF, GRF, HD ZIP, HSF, LBD, MADS, MYB, NAC, ORPHANS, SBP, TALE and WRKY TF families. Log2 modified RPKM values of DE TFs were used to generate heatmap and clusters using heat mapper resulted in four clusters (Fig. 6; Supplementary File 8). The first cluster represents the set of genes with very high expression levels all over the reproductive stages. This group contains mainly AP2/ERF, AUX/IAA, bHLH, TALE, MYB members, and a few of the NAC, DOF, MADS, and HSF members. The second cluster is represented by genes having very low expression during early developmental stages (R4 and R5) and higher expression at later stages (R6 and R7). This cluster is dominated by the bZIP, C2H2, DOF, MADS, MYB, NAC, TALE, and WRKY, and few members of HSF, bHLH, G2 like TF, HD ZIP and Trihelix TFs. The third cluster represents a group of genes that express highly at early stages (R4 and R5), and their expression reduced gradually towards pod maturation, suggesting their role in pod organization, enlargement, or metabolite biosynthesis. Important TFs represented in these clusters are AP2/ERF (mainly ERFs), ARF, ARR, bHLH, bZIP, C2H2, DOF, HD ZIP, NAC, SBP and specially MYB TFs. The fourth cluster represents TF genes with high expression at the R4 stage but reduced expression at later stages. The TFs represented in this cluster, GRF, LBD, ARF, GRAS, and MYB, suggesting their role during the initial pod development stage but not in maturation. Overall, this cluster analysis suggests the involvement of MYB, NAC, DOF, bHLH, MADS, AP2/ERF, ARF and AUX/IAA in peanut shell development, whereas AP2/ERF and MYB are involved in all development stages and NAC and WRKY at the mature stages. NAC TFs are reported as master regulators in Arabidopsis plant cell wall biosynthesis, especially SCW [69, 70]. A rice MYB transcription factor, *OsMYB58*/*63* was found to directly up-regulate the expression of a rice secondary cell wall-specific cellulose synthase gene, cellulose synthase A7 (*OsCesA7*); in contrast to this, the Arabidopsis putative orthologs *AtMYB58* and *AtMYB63* have been shown to specifically activate lignin biosynthesis [71, 72]. It can be presumed that modifications and other reorganization occurring in the shell is a similar process that occurs during secondary cell wall formation; to provide the structural rigidity and ability to withstand the attack of pathogens and mechanical wounding to save the seeds.

AP2-like transcription factor AIL1, ERF RAP2–3, ERF CRF4, ERF034, ARF18; Auxin-responsive protein IAA31, IAA26, MYB44 and Gibberellin induced genes were up-regulated in this study. Li et al. [27] reported the same result for the peg and early pod development stages. Five HSF TFs (HSF24 and HSFB group members) showed increasing expression at R6 and R7 stages. Xia et al. [23] also reported five HSF TFs to be up-regulated at the late stage, which suggests the involvement of these TFs to overcome the stress conditions during pod development. In another report, transcription factor families like WRKY, MYB, bHLH and MADS were also identified as DE in the embryo-located tip region (ER) of the developing pod during the enlargement stage [73].

Downregulation of ethylene-responsive TFs at both the R6 and R7 stages and higher expression of AUX/IAA TFs throughout the pod development process were observed (Supplementary File 8). Downregulation of ERF TFs was also observed in gynophores undergoing soil penetration and development [68, 73]. Peanut developing pod maintains lower ethylene level and higher auxin level to facilitate cell division and elongation, suggesting a basal level of ethylene might be required to maintain normal cell division and elongation [28]. Taken together, these findings suggest a strong correlation between auxin and ethylene interplay during pod development.

According to the results, we can conclude that cell wall biosynthesis in peanut shell is regulated by two-tier of TFs: the first level comprises NAC, WRKY, and MYB TFs, while the second level comprises MYB, ARF, AUX/IAA, AP2/ERF, AGL, C2H2, EF, HSF, DOF, GRF, TGA, MADS, HAT, TALE, LBD, bHLH, HD ZIP and ZF HD TFs which are under the regulation of first level TFs. In Arabidopsis, MYB TFs and NAC TFs are also the main players of the secondary cell wall regulatory network [28, 70, 74].

### Metabolite analysis of developing peanut shell

Shell metabolites analysis was performed to compare several significant constituents between Hanoch and IGC53 during pod development (Table 1). Overall fiber, crude fiber, cellulose, lignin, hemicelluloses, and dry matter were increased with shell development, while K, N, protein, and ash content were decreased. During development, the transition from R5 to R6 stage represents the most significant expansion in the seed and shell size, up to a maximum, which is reflected by a profound change in the peanut shell constituents like cellulose, crude fiber, hemicellulose and lignin (Table 1).

**Table 1:** Estimates of peanut’s shell components for genotypes Hanoch and IGC53 in three development stages R5, R6 & R7. (Neutral detergent fibre-NDF, Acid detergent fibre-ADF, Acid detergent lignin-ADL)

Shell components	Genotypes	Pod developmental stages		
		R5	R6	R7
P	Hanoch	0.23 ± 0.00	0.19 ± 0.01	0.13 ± 0.02
	IGC53	0.24 ± 0.01	0.21 ± 0.01	0.10 ± 0.00
K	Hanoch	1.50 ± 0.03	1.17 ± 0.01	0.89 ± 0.03
	IGC53	2.12 ± 0.28	2.58 ± 0.17	1.21 ± 0.09
Ca	Hanoch	0.34 ± 0.00	0.36 ± 0.01	0.39 ± 0.04
	IGC53	0.31 ± 0.03	0.45 ± 0.01	0.42 ± 0.06
ADF (%)	Hanoch	23.20 ± 1.70	46.65 ± 9.55	70.25 ± 1.45
	IGC53	29.90 ± 0.20	54.40 ± 2.00	75.50 ± 1.20
NDF (%)	Hanoch	30.25 ± 2.25	54.70 ± 7.90	79.60 ± 0.20
	IGC53	35.20 ± 1.10	64.05 ± 3.25	84.35 ± 0.15
Crude fiber (%)	Hanoch	12.20 ± 0.93	36.63 ± 6.47	59.35 ± 1.29
	IGC53	18.54 ± 1.46	45.22 ± 0.67	62.43 ± 1.05
Cellulose (%)	Hanoch	20.75 ± 1.85	33.69 ± 6.41	45.30 ± 1.20
	IGC53	25.00 ± 0.90	40.08 ± 1.88	47.25 ± 2.15
Hemicellulose (%)	Hanoch	7.05 ± 0.55	8.05 ± 1.65	9.35 ± 1.25
	IGC53	5.30 ± 0.90	9.65 ± 1.25	9.15 ± 1.35
ADL (%)	Hanoch	2.44 ± 0.16	12.96 ± 3.14	24.95 ± 0.25
	IGC53	4.88 ± 0.72	14.32 ± 0.12	27.95 ± 0.95
Ash (%)	Hanoch	11.40 ± 2.00	8.30 ± 0.60	7.80 ± 0.50
	IGC53	10.35 ± 0.15	11.75 ± 0.95	8.40 ± 0.30
Dry matter (%)	Hanoch	9.58 ± 0.60	17.24 ± 3.74	31.36 ± 3.36
	IGC53	10.25 ± 0.21	20.10 ± 1.65	36.67 ± 2.09
Protein (%)	Hanoch	17.13 ± 0.25	15.57 ± 0.53	11.47 ± 1.17
	IGC53	14.94 ± 1.39	13.66 ± 2.30	6.45 ± 0.13
N	Hanoch	2.30 ± 0.06	2.24 ± 0.01	1.66 ± 0.11
	IGC53	2.07 ± 0.18	1.74 ± 0.58	0.95 ± 0.04

As indicated from Table 1, genotype IGC53 contains a higher level of crude fiber, cellulose, NDF, ADF, ADL, K, ash, and dry matter percentage than Hanoch in all pod development stages. In contrast, higher protein and nitrogen content were observed in the shell of Hanoch. The hemicellulose content was similar for both genotypes at the R7 stage, but Hanoch had more hemicellulose at the R5 stage. The acid detergent lignin (ADL) content in genotype IGC53 was double that of Hanoch in R5 developmental stage. Ca^2+^ content increased slightly during pod development but was higher in IGC53 than in Hanoch. In addition to pectic substances, Ca^2+^ is also an important factor for shell development because it forms complexes in combination with lignin or carbohydrates through the phenolic acids in the cell wall and plays an essential role in providing stability to the cell wall of the peanut shell [13]. The results of the metabolite analysis support the transcriptomic findings observed in this study in terms of the enriched processes during the stages of shell development like cell wall biosynthesis (governed by Cellulose Synthases and cell wall modifying genes) and the secondary metabolic process specified for lignin biosynthesis genes like *Isoflavonemethyl transferase*, *Cinnamyl alcohol dehydrogenase,* and *Feruloyl hydroxylase.*

## Conclusions

This study performed a comparative transcriptome analysis to identify differentially expressed genes, enriched processes and molecular mechanisms during peanut shell development. Cell wall biosynthesis/modifications, carbohydrate metabolic process, signaling, transcription factors, transport, stress and lignin biosynthesis pathway were the important processes that were identified. The expression of the cell wall and lignin-related genes was correlated with the phenotypic and metabolite changes during the shell formation and pod expansion. TFs and other genes like chitinases were also enriched in peanut shells known for pathogen resistance against soilborne major pathogens and pod damages.

The data collected from this study will help decipher the pod development process and assist in identifying target candidate genes for shell organization and disease resistance for future breeding and research purposes. For example, in the pod wart disease, the resistance of IGC53 may be conferred by modifying specific cell wall components. Pod wart is characterized by unsightly necrotic warts or scabs caused by soil-borne bacteria from the *Streptomyces* genus. The streptomyces attack the shell surface early in the pod development, and the cell wall composition controls their progression. Therefore, modification of target genes for early cell wall biogenesis and cell wall modification process may be used to facilitate molecular breeding for resistance.

## Methods

### Plant materials and tissue collection

Hanoch (*A. hypogaea* ssp. *hypogaea* var. *hypogaea*) and genotype IGC53 (the local name for PI338338) (*A. hypogaea* ssp. *fastigiata* var. *peruviana*), two peanut genotypes were used for this analysis. ‘Hanoch’ is a Virginia-type cultivar with a bright yellow shell color and smooth skin that has a relatively high susceptibility to pathogens associated with pods. Genotype IGC53 is a Peruvian peanut-type with a highly reticulated pod surface and relatively high tolerance to diseases like pod wart and pod netting [75] but with low pod-filling potential [76]. Genotype Hanoch was developed by ARO, Israel, and seeds of IGC53 were obtained from USDA, station Griffin, GA, USA. Seeds for both the genotypes were propagated by Hovav lab, ARO, Israel. The experimental plant research has complied with institutional and national guidelines. Field studies were conducted under local legislation (permission by the Israeli Ministry of Agriculture). In a randomized block experimental system with three blocks, plants were grown in the field at the Israel Ministry of Agriculture Southern R & D Center, Negev, Israel; as defined by Gupta et al. [76]. At 110 days post sowing, plants from all six plots (2 genotypes × 3 repetitions) were uprooted and pods were collected manually. According to Boote [8], the pods were then sorted into four developmental stages (R4, R5, R6 and R7) (Fig. 1). The shells were separated from seeds, flash-frozen in liquid nitrogen, and stored at − 80 °C till RNA extraction. The seeds were used for a previous transcriptome analysis, reported by Gupta et al. [43], while shells from the same samples were used for the current study.

### Isolation of total RNA, RNA-Seq library preparation and sequencing

400 mg from each ground sample were used for RNA extraction using the Hot Borate method described by Brand and Hovav [77]. The total RNA’s integrity was analyzed on an agarose gel, and its quality was assayed using Nanodrop ND-1000. Four μg of the total RNA was used for the preparation of RNA-Seq libraries using TruSeq RNA Sample Preparation Kit v2 (Illumina) following the Manufacturer’s protocol. With 4 developmental stages × 2 peanut genotypes × 3 biological replicates, a total of 24 libraries were constructed. RNA-Seq libraries were validated by DNA Screen Tape D1000 using the Tapestation 2200 (Agilent Technologies), quantified by Qubit, normalized and sequenced by an Illumina HiSeq™ 2000.

### Analysis of RNA-Seq data

Raw reads were cleaned using the FASTX Toolkit (http://hannonlab.cshl.edu/fastxtoolkit/index.html, version 0.0.13.2) and read with the quality scores with less than 70% base pairs with quality score ≤ 30 was filtered out. The transcript expression was performed with Bowtie 2 [78] using the genome-guided tetraploid peanut transcript assembly (http://www.peanutbase.org/). The expression was also obtained as Reads per Kilobase per Million mapped reads (RPKM) values.

### Differentially expressed (DE) and functional gene analyses during shell development

Read counts of the gene expression were subjected for DE expression analysis in R software v.3.2.0 (R Foundation for Statistical Computing, Vienna, Austria) using DESeq2 [78]. Differential expression of genes was measured between a single genotype’s neighboring developmental stages and between the two genotypes at the same developmental level, controlled by false discovery rate (FDR) using the BH method [79] at α = 0.05. Normalized RPKM values were extracted by using a combined list of all the differentially expressed (DE) genes between genotypes at different developmental stages. Normalized RPKM (log_2_ values) of gene expression was used to generate heatmap and hierarchical clustering in R using the gplots (http://www.rproject.org) in both genotypes separately. A transition matrix of gene expression clusters was created by comparing the expression clusters of Hanoch with clusters of IGC53 and identify functional processes in each cell using the GO enrichment tool of the Blast2GO [80] Fisher exact test controlled by Qvalue < 0.05.

### Identification and expression analysis of cellulose synthase superfamily and other cell wall biosynthesis-related genes in peanut

Genes belonging to the cellulose synthase superfamily, including cellulose synthase (*CesAs*) and cellulose synthase-like genes (*Csls*) were identified from the peanut reference genome. Their expression was analyzed during the stages of shell development using Log_2_ RPKM values obtained from RNA seq data. Apart from the cellulose synthase superfamily, other gene families involved in cell wall biosynthesis (i.e. chitinase, expansins, pectinesterase, etc.) were also selected. Their expression was analyzed during the shell development stages in both genotypes.

### Identification and study of TFs expression and shell development in peanut

Peanut TF genes were identified by GO terms and an annotation list of the peanut genome assembly. TFs were grouped into families, and log_2_ RPKM values were used to study their expression during shell development. TFs specific to shell development were identified from the DE gene list from both genotypes.

### Peanut shell component analysis

Peanut shell samples were prepared in replicates from R5, R6, and R7 developmental stages for both Hanoch and IGC53 genotypes; each sample was a mix of ten pods selected randomly from the plot. The samples were dried at 60 °C in a hot air oven and crushed in a coffee blender. The crude fiber, cellulose, hemicellulose, acid detergent lignin (ADL), neutral detergent fiber (NDF), and acid detergent fiber (ADF) content were determined by ANKOM’s fiber analyzer F200 (ANKOM Technology Corporation, Fairport, NY). Protein and nitrogen content were estimated by Nessler’s reagent [81]. Phosphorus was assessed by spectrophotometry, while calcium and potassium were analyzed by flame photometry. Furthermore, the ash and dry matter content were measured by Filter Bag Technique (for A200 and A200I) [82] (Supplementary File 9).

### Permissions and/or licenses for the collection of plant or plant samples

All methods used in the study complied with relevant institutional, national, and international guidelines and legislation.

## Supplementary Information

**Additional file 1: Supplementary Fig. 1**: Differential expression matrix and enriched processes between Hanoch and IGC53 genotypes. The matrix is arranged according to four major gene expression clusters in each genotype. Each cluster is compared with the four clusters of the other genotype. Values in each cell represent the number of shared genes between respective clusters in both genotypes. H1 = Hanoch cluster 1; H2 = Hanoch cluster 2 etc. Cells colored in red represent situations where Hanoch is expressed higher than IGC53; cells in green represent higher expression in IGC53 than Hanoch; Blue colored cells represent similar expression in Hanoch and IGC53.

**Additional file 2: Supplementary File 1**: GO enrichment during pod development processes of successive developmental stages in both genotypes’ peanut shells.

**Additional file 3: Supplementary File 2**: Significant enrichment of GO terms in cluster matrix presented in a 4 × 4 transition matrix.

**Additional file 4: Supplementary File 3**: Differentially expressed TF between genotypes present in H-2:53–1 cluster matrix.

**Additional file 5: Supplementary File 4**: Details and sequence of total of 155 genes of the cellulose synthase superfamily.

**Additional file 6: Supplementary File 5**: Log_2_ transformed RPKM expression of CES superfamily was analyzed during different shell development stages.

**Additional file 7: Supplementary File 6**: Log_2_ transformed RPKM expression data for cell wall modification-related genes and cluster analysis.

**Additional file 8: Supplementary File 7**: Log_2_ RPKM expression of TFs belonging to 67 TF families.

**Additional file 9: Supplementary File 8**: Log_2_ modified RPKM values of DE TFs to generate heatmap and clusters using heat mapper resulted in four clusters.

**Additional file 10: Supplementary File 9**: Procedure used for metabolite analysis of peanut shell.

## Data Availability

The datasets used in the current study are available from the corresponding author on reasonable request. Sequences have been deposited in NCBI Sequence Read Archive under Bioproject PRJNA732586. (https://www.ncbi.nlm.nih.gov/bioproject/732586).

## Abbreviations

ADL: The acid detergent lignin
BRU1: Brassinosteroid regulated protein
CesAs: Cellulose synthase A
CHI: Chitinase
CL: 4-Coumarate-CoA ligase 1
CCR: Cinnamoyl-CoA reductase 1
COMT: Caffeoyl-CoA O-methyltransferase
Csls: Cellulose synthase-like genes
DE: Differentially expressed genes
FDR: False discovery rate
GAX: Glucurono arabinoxylan
GO: Gene Ontology
RLKs: G-type lectin S-receptor-like serine/threonine-protein kinases
RPKM: Reads per kilobase million
TF: Transcription factor
XET: Xyloglucan Endotransglucosylase
XGT: Xyloglucan Galactosyltransferase
XI: Xylose Isomerase
XK: Xylose Kinase
XPS: Xylose proton symporter
XT: Xylose transferase
XTH: Xylosyltransferase Hydrolase
