# Supplementary figures and images for: Deep transcriptomic study reveals the role of cell wall biosynthesis and organization networks in the developing shell of peanut pod
Source: BMC Plant Biol. 2021 Nov 3;21:509. doi: 10.1186/s12870-021-03290-1 (PMC8565004; doi:10.1186/s12870-021-03290-1)

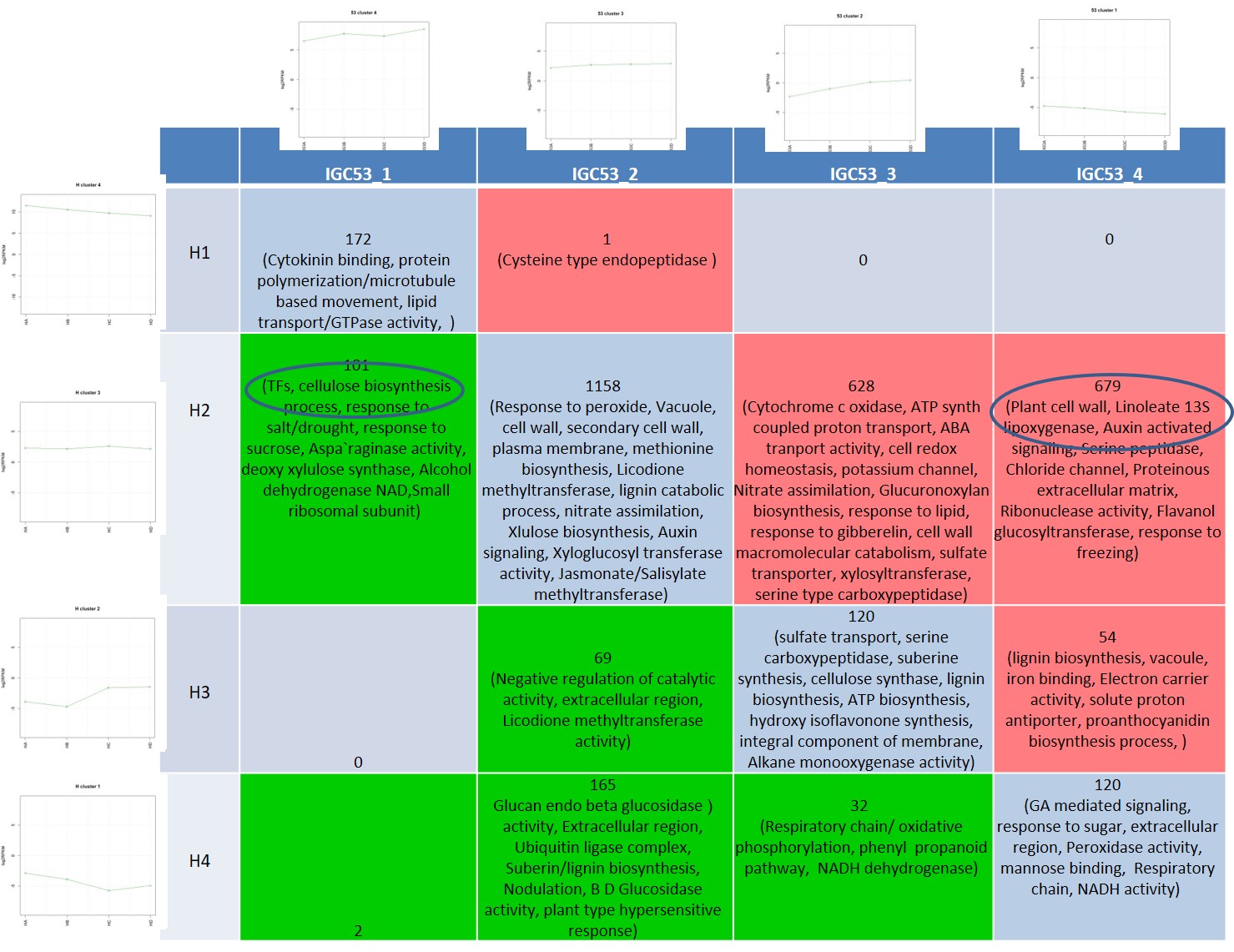

Supplement: Supplementary file 1 — Additional file 1: Supplementary Fig. 1: Differential expression matrix and enriched processes between Hanoch and IGC53 genotypes. The matrix is arranged according to four major gene expression clusters in each genotype. Each cluster is compared with the four clusters of the other genotype. Values in each cell represent the number of shared genes between respective clusters in both genotypes. H1 = Hanoch cluster 1; H2 = Hanoch cluster 2 etc. Cells colored in red represent situations where Hanoch is expressed higher than IGC53; cells in green represent higher expression in IGC53 than Hanoch; Blue colored cells represent similar expression in Hanoch and IGC53. [file 12870_2021_3290_MOESM1_ESM.jpg]
