# Supplementary material for: Deep transcriptomic study reveals the role of cell wall biosynthesis and organization networks in the developing shell of peanut pod
Source: BMC Plant Biol. 2021 Nov 3;21:509. doi: 10.1186/s12870-021-03290-1 (PMC8565004; doi:10.1186/s12870-021-03290-1)
Supplement: Supplementary file 10 — Additional file 10: Supplementary File 9: Procedure used for metabolite analysis of peanut shell. [file 12870_2021_3290_MOESM10_ESM.docx]

**Title:** **Deep transcriptomic study reveals the role of cell wall biosynthesis and organization networks in the developing shell of peanut pod**

Kapil Gupta^a#^ *, Shubhra Gupta^a^, Adi Faigenboim-Doron^a^, Abhinandan Surgonda Patil^a$^, Yael Levy^a^, Scott Cohen Carrus^a^, Ran Hovav^a^ *

^a^ Department of Field Crops, Plant Sciences Institute, ARO, Bet-Dagan, Israel.

# Current Address: CSIR-Central Institute of Medicinal and Aromatic Plants, Lucknow, UP, India.

$ Current Address: Plant Breeding Strategic Innovation Platform, International Rice Research Institute, Los Banos, Laguna, Phillipines.

* Communicating Author

E-mail address: ranh@volcani.agri.gov.il (Ran Hovav)

kapilgupta190184@gmail.com (Kapil Gupta)

Supplementary Table 1. Details for detection methods used in estimation of shell components.

| Standard method | Description of the test | Name of the test | Shell components | Sr.No. |
| --- | --- | --- | --- | --- |
| SM 4500 P-E | The test is based on a color reaction, in which a complex of phosphorus is formed | Examination of phosphorus concentration in the water | P-Phosphorus | 1 |
| SM 3500 K-B | Tested using a flame photometer | Test potassium concentration in the water | K-Potassium | 2 |
| SM 3111 B | Tested by evaporating the sample into a flame and reading the amount of light absorbed by each element at the appropriate wavelength | Measuring the presence of Ca element using an atomic absorption device in the water | Ca-Calcium | 3 |
| ANCOME Technology | Cellulose, Hemi-celluloseare calculated from ADF ,NDF and ADL  (https://www.ankom.com/analytical-methods-support/fiber-analyzer-a200) | ADF, NDF,Crude fiber, Cellulose, Hemi-cellulose | ADF (%), NDF (%)Crude fiber (%), Cellulose (%), Hemi-cellulose (%) and ADL (%) | 4 |
| Filter Bag Technique (for A200 and A200I) | The principle of the method for testing dry matter is based on drying the known weight of an example in the oven and removing the water from it by evaporation. % Dry matter is calculated as the remainder of the remaining material from the wet sample rule. In general, evaporation of water from the example takes place at a temperature of 105°C.Ash is tested after dry mater in 550°C for 8 hours. | Ash and dry matter | Ash and Dry matter (%) | 5 |
| Nessler's reagent | An aqueous solution of potassium iodide, mercuric chloride, and potassium hydroxide, used as a test for the presence of ammonia. | Protein and Nitrogen | Protein (%)  and Nitrogen | 6 |
